# Exposure to Environmental Tobacco Smoke (ETS) among Employees of Hospitality Venues in the Light of Changes in Anti-Tobacco Legislation in Poland

**DOI:** 10.3390/ijerph17103691

**Published:** 2020-05-23

**Authors:** Emilia Krakowiak, Katarzyna Sygit, Marian Sygit, Elżbieta Cipora, Jan Krakowiak

**Affiliations:** 1 Social Medicine Unit, Department of Social and Preventive Medicine, Medical University of Łódź, 90-647 Łódź, Poland; emilia.krakowiak@wp.pl (E.K.); jan.krakowiak@umed.lodz.pl (J.K.); 2 Faculty of Health Sciences, President Stanislaw Wojciechowski State Vocational Academy in Kalisz, 62-800 Kalisz, Poland; msygit@onet.pl; 3 Sanok Medical Institute, State Vocational Academy in Sanok, 38-500 Sanok, Poland; elacipora@interia.pl

**Keywords:** smoking, tobacco smoke, exposure to tobacco smoke, ETS, anti-tobacco law, hospitality venues

## Abstract

*Introduction:* Numerous studies conducted in Europe and worldwide have indicated that employees of hospitality venues are the most exposed professional group to environmental tobacco smoke (ETS) in the workplace. The purpose of this study was to assess the exposure of employees of hospitality venues to ETS in the light of changes in anti-tobacco legislation in Poland. *Materials and methods:* The study consisted of two stages. The first stage was conducted in 2010, while the second in 2015. The study was conducted among employees of 300 randomly selected hospitality venues in the city of Łódź (Poland). In total, 2607 questionnaires were analysed. The study used two survey questionnaires created and recommended by the Institute for Global Tobacco Control to study exposure to ETS. Statistical analysis was made with Statistica 13.1 PL (StatSoft, Poland). *Results:* In the group of all nonsmoking employees, individuals exposed to ETS at work in 2010 accounted for 72.6%; while in 2015 it was 51.8%. Factors affecting exposure to ETS in the workplace included, among others: age, marital status, education, position held, presence of a smoking room on the premises, and noncompliance with the provisions of the anti-tobacco laws. *Conclusions:* The prevalence of tobacco smoking among employees of hospitality venues decreased in 2010–2015, however, it remained high. More than half of nonsmoking employees were exposed to ETS at work.

## 1. Introduction

Environmental tobacco smoke (ETS) is the sum of second-hand smoke (SHS) and third-hand smoke (THS). SHS is the combination of the side-stream cigarette smoke in the intervals between puffs as a result of cigarette smouldering and the smoke exhaled by the smoker, while THS is the smoke persisting in the environments long after the active smoking is ceased. Thus, second-hand smoke exposure consists of an unintentional inhalation of smoke that occurs close to people smoking and/or in indoor environments where tobacco was recently used, while THS exposure occurs in enclosed environments where tobacco was used hours or days before [1,2].

Exposure to ETS has serious health consequences. The increased risk of developing malignant neoplasms, noncancer respiratory diseases, cardiovascular diseases, or pregnancy failure needs to be highlighted. Due to numerous health threats, promotional and educational activities, as well as new legal solutions, to protect nonsmokers against passive exposure to ETS have been introduced for many years [3,4,5,6].

In 1998, the state of California was the first in the United States to introduce comprehensive legal solutions prohibiting smoking in all workplaces, including bars and restaurants, and in 2004, Ireland was the first European country to introduce a total ban on smoking in public places [7,8,9,10,11,12].

Legal measures introduced in other European countries differ in terms of the degree of limitation of exposure to ETS—ranging from a total smoking ban in all workplaces (including hospitality venues) to partial protection by means of creating designating areas in bars or restaurants for smokers (separate rooms where smoking is allowed) [13,14,15,16,17].

Numerous studies conducted in USA, Europe, and elsewhere indicate that it is the employees of bars, cafes, restaurants, nightclubs, and music venues that are the most exposed professional group to ETS in the workplace; therefore, this group desperately needs legal protection of their health interests [18,19,20,21,22,23].

In recent years, a very large impact on shaping tobacco policy in Poland has been made by the European Parliament and the European Council. The Polish Anti-Tobacco Act (Act of July 22, 2016, amending the Act on Protection of Health against the Consequences of Use of Tobacco and Tobacco Products, Journal of Laws 2016, item 1331) in its current form contains many provisions aimed at protecting life and health against the consequences of exposure to ETS [17,24,25].

In Poland, the Anti-Tobacco Act (Act of July 22, 2016, amending the Act on Protection of Health against the Consequences of Use of Tobacco and Tobacco Products, Journal of Laws 2016, item 1331), despite its restrictions on exposure to ETS, does not fully protect from such exposure [14,17].

Unfortunately, the Polish legislator, despite many amendments made to the Act on Health Protection against the Consequences of Use of Tobacco and Tobacco Products, did not decide to introduce an absolute ban on smoking in hospitality venues. It means that these establishments are not completely smoke-free and safe for employees and customers alike [17,21,26].

Another aspect of tobacco law is their proper implementation, compliance, and enforcement. Unfortunately, in many hospitality venues, smoking ban is frequently violated, often with the indifferent attitude of employers, venue owners, staff, and even people exposed to ETS [10,27].

Importantly, according to WHO’s recommendations, the most effective protective measures against exposure to ETS are only those that lead to complete elimination of smoking and tobacco smoke, while all other technical solutions, such as ventilation, air filtration, separate smoking rooms, do not provide complete protection for nonsmokers [3,15,19]. Researches conducted in countries such as Ireland, Norway, Scotland, and France, whose authorities have decided to introduce a total ban on smoking in bars, restaurants, and clubs, confirm the decrease in employees’ exposure to ETS, improvement of their health, and thus, the effectiveness of such strict tobacco laws. In these countries, legal changes were accompanied by anti-tobacco social campaigns to make people aware of the seriousness of the problem of exposure to ETS and of health benefits brought by a total ban on smoking in public places [1,2,28,29].

The new legislative solutions are therefore aimed at protecting individuals from exposure to ETS in public places and protect employees of hospitality venues against such exposure. It is important, however, that only a full ban on smoking in workplaces (hospitality venues) can effectively protect employees from exposure to ETS [3,5,7].

The purpose of this study was to assess the exposure of employees of hospitality venues to ETS in the light of changes in anti-tobacco legislation in Poland.

## 2. Materials and Methods

The study consisted of two stages. The first stage was conducted in 2010 among 1360 employees of 300 randomly selected hospitality venues in the city of Łódź (Poland). The second stage was conducted in 2015 in the same establishments. It should be emphasized that in 2015, out of 300 premises included in the first study, 57 had been closed. Thus, the second stage of the study was conducted among 1247 employees from 243 hospitality venues in Łódź.

To collect empirical data, the study used two survey questionnaires created and recommended by the Institute for Global Tobacco Control to study exposure to ETS.

The survey questionnaire used in the first stage of the study contained 41 questions. It covered basic demographic and work-related questions, as well as questions about smoking and exposure to ETS at work, at home, and in other public places. The respondents were also asked about their smoking behaviours, exposure to ETS, and their health. The 2015 survey questionnaire contained 86 questions and was extended for the purposes of the second stage with additional questions to enable inter alia assessment of the functioning of the amended anti-tobacco law, opinion on the harmfulness of the use of regular and smokeless tobacco, and opinion on the introduction of the smoking ban in various situations and various places. Both survey questionnaires were anonymous and voluntary, intended for self-completion by respondents.

Our epidemiological study, which assessed the exposure to ETS among employees of hospitality venues in the light of changes in anti-tobacco legislation in Poland, is one of the first such large population studies to be carried out in Poland using two questionnaires created and recommended by the Institute for Global Tobacco Control for ETS exposure testing.

The use of the questionnaire-survey recommended for assessing exposure to ETS and treated as diagnostic tests in epidemiological studies was verified in pilot studies which confirmed the credibility, reliability, and validity of the results obtained. They were assessed by experts as appropriate for use in this epidemiological study. Assessment of ETS exposure is also performed in clinical-laboratory studies by testing u-cotinine in urine.

However, in epidemiological and population studies (such as our study), a good research tool is the diagnostic test used by us and recommended by the Institute for Global Tobacco Control, which helps achieve the intended research goal.

In total, 2607 questionnaires were analysed. The project of the study received a positive opinion from the Bioethics Committee at the Medical University of Łódź (no. RNN/117/15/KE of April 21, 2015)

Statistical analysis was made with Statistica 13.1 PL (StatSoft, Poland).

The structure of the study groups analysed according to variables was described with structural indicators (%). To test the relationship between the analysed variables, a χ^2^ test was used.

Due to the size of groups in individual analyses, the test was modified with *Yates’s correction for continuity.*

Single-factor and multifactor regression models were used in the statistical analysis. Single-factor logistic regression allowed to assess the relationship between exposure to ETS (dependent variable) and selected demographic and social characteristics, occupation, and working conditions declared by the respondents (independent variables). Independent variables were also included in the statistical analysis using a multifactor regression model to assess their simultaneous impact on the dependent variable.

Additionally, the odds ratios (OR) were calculated with their corresponding 95% confidence intervals (95% CI). The odds ratio is the ratio of a chance of occurrence of a given phenomenon in one group to the chance of its occurrence in another group. OR ≈ 1 means that the odds are similar, OR < 1 means that the chance of occurrence in the study group is smaller than in the reference (control) group, while OR > 1 means that the chance is greater.

## 3. Results

### 3.1. The Overview of the Subjects

In the 2010 study group of 1360 individuals, 17.2% of respondents (234 people) were aged 20 and less, 63.7% (867 people) were aged 21–30, while 13.1% of respondents (178 persons) were aged 31–40. Among 1247 respondents of the 2015 survey, the percentage of people aged 20 and less was 10.9% (136 people), 67.2% (838 people) were aged 21–30, and 14% of respondents (175 people) were aged 31–40. There was no statistically significant difference in the structure of respondents by age in 2010 and 2015 (*p* > 0.05).

In the group of 1360 subjects in 2010, 34.9% of subjects (475 people) were men, while 65.1% (885 people) were women.

Among 1247 people participating in the 2015 survey, the proportion of men was 35% (437 people), while 65% of respondents (810 people) were women. There was no statistically significant difference in the structure of respondents by gender in 2010 and 2015 (*p* > 0.05).

In the 2010 study group of 1360 individuals, 1.2% of respondents (17 people) completed primary education, 10.2% (139 people) completed vocational education, while 59.6% (810 people) completed secondary education. Higher education, bachelor’s or master’s degree, was declared by 29% of respondents (394 people).

Among 1247 respondents of the 2015 survey, the percentage of people with primary education was 1.2% (15 people), 10% (125 people) reported vocational education, while 58.8% of respondents (733 people) declared secondary education. In particular, 30% of respondents (374 people) declared higher education, Bachelor’s or Master’s degree. There was no statistically significant difference in the structure of respondents by education in 2010 and 2015 (*p* > 0.05).

In the 2010 study group of 1360 respondents, 73.5% of them (999 people) declared being single, 24.6% (334 people) were married, 1.2% of respondents (16 people) were divorced, while 0.8% of respondents (11 people) declared to be a widow or a widower.

Among 1247 participants of the 2015 survey, 45.4% (566 people) declared to be single, 39.9% (498 people) were married, 13.3% of respondents (166 people) were divorced, while 1.4% of respondents (17 people) reported being a widow or a widower. The observed differences were statistically significant (*p* < 0.001; chi^2^ = 272.655) (Table 1).

### 3.2. Analysis of Respondents’ Answers on Tobacco Smoking

In 2010, in the study group of 1360 respondents, 24.9% of them (339 people) reported smoking on daily basis, while 10.4% (141 people) declared occasional smoking. Nonsmokers accounted for 64.7% (880 people). In 2015, in the study group of 1247 respondents, 23% of them (287 people) reported smoking on daily basis, while 6.7% (83 people) declared occasional smoking. Nonsmokers accounted for 70.3% (877 people). The observed differences were statistically significant (*p* < 0.001; chi^2^ = 14.472).

In 2010, in a group of 480 smokers, 25.4% of respondents (122 people) said that they smoked 10 or fewer cigarettes a day, 51.9% of respondents (249 people) stated that they smoked from 11 to 20 cigarettes a day, 1% of respondents (5 people) smoked between 21 and 30 cigarettes per day, 0.2% of respondents (1 person) smoked 31 or more cigarettes a day, while 21.5% of respondents (103 people) chose ‘I don’t know’ as their response.

In 2015, in a group of 370 smokers, 46% of respondents (170 people) said that they smoked 10 or fewer cigarettes a day, 31% of respondents (115 people) stated that they smoked from 11 to 20 cigarettes a day, 22.2% of respondents (82 people) smoked between 21 and 30 cigarettes per day, while 0.8% of respondents (3 people) smoked 31 or more cigarettes a day. The observed differences were statistically significant (*p* < 0.001; chi^2^ = 212.883).

In 2010, in a group of 1360 respondents, 31.1% (423 people) declared working in a smoking room, 37.6% (511 people) in a nonsmoking room, and 31.3% (426 people) respondents reported working in both rooms. In 2015, in the group of 1247 respondents, 4.7% (59 people) declared working in a smoking room, 73.5% (917 people) in a nonsmoking room, and 21.7% (271 people) respondents reported working in both rooms. The observed differences were statistically significant (*p* < 0.001; chi^2^ = 420.681).

In 2010, in the study group of 1360 people, 96.5% (1312 people) declared that the smoking ban in the nonsmoking room was observed, while 3.5% (48 people) said that the smoking ban was not observed. In 2015, in a group of 1247 respondents, 88.5% (1104 people) declared that the smoking ban in the nonsmoking room was observed, while 11.5% (143 people) said it was not observed. The observed differences were statistically significant (*p* < 0.001; chi^2^ = 60.374).

In the group of 1360 respondents in 2010, 89.9% of respondents (1222 people) declared that there was a room for smokers in their establishment. Among 1247 participants of the 2015 survey, affirmative answer to this question was given by 56% (698 people). The observed differences were statistically significant (*p* < 0.001; chi^2^ = 384.715).

In the group of 880 nonsmokers surveyed in 2010, 49% (431 people) reported that they stayed in a room for smokers in their workplace every day, 8.5% (75 people) declared such stay once a week, 15.1% (133 persons) once a month, while 27.4% of respondents (241 persons) replied that they never stayed in such rooms.

In the group of 877 nonsmokers surveyed in 2015, 25% (219 people) reported that they stayed in a room for smokers in their workplace every day, 10.3% (90 people) declared such stay once a week, 16.5% (145 people) once a month, while 48.2% of respondents (423 people) replied that they never stayed in such rooms. The observed differences were statistically significant (*p* < 0.001; chi^2^ = 120.907) (Table 2).

### 3.3. Impact of Selected Factors which Expose Hospitality Venues Staff to ETS in the Workplace in 2010 and 2015—Single-Factor and Multifactor Analysis

In the group of all nonsmoking employees, individuals exposed to ETS at work in 2010 accounted for 72.6% (639 people). The one-factor logistic regression analysis shows that factors affecting exposure to ETS at work in 2010 included: age, marital status, education, position held, presence of the smoking room, noncompliance with the anti-tobacco laws, and the type of room in which a respondent worked.

The largest increase in the odds ratio of exposure to ETS at work was observed among bartenders/waiters (OR 72.29; 95% CI 17.30–30.20; *p* < 0.001) and cooks (OR 55.45; 95% CI 13.06–235.46; *p* < 0.001).

The highest exposure to ETS at work was reported in the group of people under 20 years old (OR 7.87; 95% CI 3.48–17.82; *p* < 0.001) and in the group of people with primary and vocational education (OR 17.76; 95% CI 5.42–58.17; *p* < 0.001).

A significant increase in the odds ratio of exposure to ETS was observed among respondents working in the smoking rooms (OR 6.80; 95% CI 4.38–10.54; *p* < 0.001).

The analysis also showed a significant increase in the odds ratio of exposure to ETS at work in a group of people whose workplace did not respect provisions of the anti-tobacco law (OR 5.89; 95% CI 1.39–24.88; *p* < 0.001) (Table 3).

In the group of all nonsmoking employees, individuals exposed to ETS at work in 2015 accounted for 51.8% (454 people). The one-factor logistic regression analysis showed that factors affecting exposure to ETS in the workplace in 2015 included: whether anti-tobacco laws were followed, whether there was a smoking ban outside the room intended for customers and whether these provisions were observed, type of venue, working hours, the rules regarding smoking in the workplace, whether there was a smoking room in the establishment, whether the venue had its own regulations prohibiting the use of tobacco products by employees indoors, whether these regulations had been communicated to employees, whether the venue had regulations prohibiting the use of tobacco products by clients indoors, awareness that exposure to ETS might cause heart disease in nonsmokers, and awareness that exposure to ETS might cause cancer in nonsmokers.

The largest increase in the odds ratio of exposure to ETS at work was observed among employees of venues where there was a room for smokers and nonsmokers (OR 42.74; 95% CI 19.24–94.97; *p* < 0.001) and among employees of establishments where smoking was allowed in all indoor rooms (OR 9.68; 95% CI 4.54–20.65; *p* < 0.001) and at a designated place (OR 3.48; 95% CI 2.33–5.19; *p* < 0.001).

A significant increase in the odds ratio of exposure to ETS was also observed among people working in establishments where smoking was not prohibited outside the smoking room intended for customers (OR 18.64; 95% CI 8.06–43,08; *p* < 0.001) and in venues whose regulations on smoking ban in rooms other than the room intended for customers were not observed (OR 6.27; 95% CI 3.72–10.58; *p* < 0.001).

The results of the analysis showed a significant increase in the odds ratio of exposure to ETS among people working in night clubs and music clubs (OR 3.63; 95% CI 1.96–6.75; *p* < 0.001) and working after 6:00 p.m. (OR 2.41; 95% CI 1.66–3.49; *p* < 0.001).

The analysis also showed a significant increase in the odds ratio of exposure to ETS at work in establishments where there was a smoking room (OR 3.84; 95% CI 2.90–5.08; *p* < 0.001).

A significant increase in the odds ratio of exposure to ETS was also observed among people working in establishments where the provisions of the anti-tobacco law were not followed (OR 6.90; 95% CI 3.49–13.63; *p* < 0.001) and in establishments which did not have regulations prohibiting the use of tobacco products by employees (OR 3.27; 95% CI 1.57–6.79; *p* < 0.001) and customers (OR 5.29; 95% CI 2.31–12.11; *p* < 0.001) indoors.

Higher exposure to ETS occurred among workers who disagreed with the statement that exposure to ETS caused heart disease (OR 3.63; 95% CI 2.11–6.24; *p* < 0.001) and cancer in nonsmokers (OR 3.78; 95% CI 2.33–6.13; *p* < 0.001) (Table 4).

A multifactor logistic regression analysis conducted in 2010 shows that the odds ratio of exposure to ETS among employees of hospitality venues was increased the most by noncompliance with the provisions of the anti-tobacco laws (OR 24.73; 95% CI 3.54–172.88; *p* < 0.01).

A significant increase in the odds ratio of exposure to ETS was observed among employees with primary, vocational, and secondary education (OR 4.78; 95% CI 3.10–7.37; *p* < 0.001) and among those working in a smoking room (OR 11.10; 95% CI 6.50–18.94; *p* < 0.001) (Table 5).

A multifactor logistic regression analysis carried out in 2015 showed that the odds ratio of exposure to ETS among employees of hospitality venues was increased the most when the establishment had both smoking and nonsmoking rooms (OR 10.88; 95% CI 3.94–30.03; *p* < 0.001).

A significant increase in the odds ratio of exposure to ETS was observed among employees of establishments that did not have regulations prohibiting the use of tobacco products by employees indoors and among employees who were unaware of whether such regulations were in force (OR 5.11; 95% CI 1.99–13.15; *p* < 0.001).

A factor that significantly increased the odds ratio of exposure to ETS was the lack of knowledge that exposure to ETS caused cancer in nonsmokers (OR 7.95; 95% CI 3.64–17.34; *p* < 0.001) (Table 6).

## 4. Discussion

The issue of exposure of hospitality venues staff to ETS at work is a very important social and health problem, directly related to the lack of full legal protection for nonsmokers [5,18,28,29,30].

In 2010, the percentage of nonsmokers exposed to ETS in the workplace was over 72%, while in 2015 it was close to 52%. The analysis of the results obtained from the studies carried out in 2010 and 2015 confirmed a significant decrease in the exposure to ETS and existence of a significant problem in terms of the exposure of hospitality venues employees to ETS.

In 2010, nonsmoking women declared more frequent exposure to ETS at work, while in 2015 it was nonsmoking men who were more likely to be exposed. Therefore, it should be stated that hospitality venues are a unique type of space where exposure to ETS is very high and this threat applies to both women and men [8,9,10].

Despite the introduction of laws prohibiting smoking in hospitality venues, in 2015, more than half of nonsmoking staff stayed in rooms where cigarettes were smoked, and therefore, were exposed to ETS at work. Comparing the results of this study with the results of the GATS study (Global Adult Tobacco Survey), employees of hospitality venues were more often exposed to inhalation of ETS (environmental tobacco smoke) than those participating in the study in the years 2009–2010 [14,31,32]. Over 33% of them declared exposure to inhalation of ETS in the last month indoors at work. Additionally, respondents of the GATS survey declared exposure to ETS: over 98% in bars, pubs, music clubs and discos, and almost 54% in restaurants, cafes, and bistros [24,32]. When analysing results of studies presented in 2011, 2013, 2015, and 2017 in reports from a nationwide survey on smoking attitudes, it may be noticed that the percentage of people exposed to ETS in hospitality venues regularly decreased in 2011–2017 [21,33]. In 2015, 16% of respondents stated that they were exposed to ETS in bars and pubs, while in 2017, only 12% of them were exposed. In cafes, these values were 9% and 6%, respectively, while in restaurants, the percentage did not decrease and continued to be 7% [24,27].

In discos and music clubs, the proportion of respondents exposed to ETS also dropped from 15% (in 2009) to 9% (in 2017) [14,34,35,36]. The presented results showed that employees of hospitality venues were significantly more often exposed to ETS at work than persons participating in the study conducted by Bogdanovica et al. [5]. Results of studies carried out in other European countries after the introduction of complete ban on smoking in hospitality venues show that the percentage of employees exposed to ETS significantly decreased [1,37,38,39,40,41].

In Ireland, new regulations helped reduce the incidence of ETS in restaurants from 85% to 3%, and in bars and pubs from 98% to 5%, whereas the cotinine level in the saliva of nonsmokers working in bars and restaurants fell by around 80% [3,19].

In Scotland, cotinine levels decreased in the saliva of nonsmoking bar and restaurant staff by 89% [42]. Studies in France also confirmed high effectiveness of new laws. There was a significant decrease in the incidence of ETS, i.e., in bars from 95.9% to 3.7% and in restaurants from 64.7% to 2.3%. The next stage of the study conducted in 2012 found out that the occurrence of ETS in bars was at the level of 6.6%, while in restaurants, it was at the level of 1.4% [19]. Finland is another positive example of the implementation of anti-tobacco laws. In this country, after the introduction of the restrictive anti-tobacco law, the number of restaurant employees who were not exposed to the ETS increased from 54% to 82%, while the number of unexposed employees of bars and pubs increased from 10% to as much as 70% [34]. The changes observed in selected countries by other authors differ significantly from the results obtained in this study. In the above-mentioned countries, there was a much greater decrease in the exposure of hospitality venues staff to ETS at work. It results from a comprehensive anti-tobacco legislation, introducing a total and absolute ban on smoking in public places, including hospitality venues. The degree of employee exposure to ETS is also influenced by the type of room in which they work [19,23,34,42].

In our survey conducted in 2010, over 31% of respondents said that they worked in a smoking room or in both a smoking and nonsmoking room, while the percentage of staff working only in the nonsmoking room was 37.6%. However, in the study conducted in 2015, there was a significant increase in the percentage of employees (over 73%) of hospitality venues who claimed to work only in a nonsmoking room. A multifactor logistic regression analysis carried out in 2015 showed that the odds ratio of exposure to ETS among employees of hospitality venues was increased the most when the establishment had both smokers and nonsmokers rooms. The results confirm partial implementation of the new tobacco laws. As a result of the ban on smoking in hospitality venues that was introduced at the end of 2010, there was a decrease in the number of bars, cafes, restaurants, and night and music clubs with rooms excluded from the smoking ban. However, in 2015, the percentage of staff who worked in smoking rooms was 4.7%, while 21.7% of staff worked both in smoking and nonsmoking rooms, which clearly indicates that the almost 5-year period of smoking ban did not completely eliminate the problem of smoking rooms. A significant impact on the incomplete implementation of the ban is a possibility left by the legislator to let owners or managers of venues with at least two rooms, i.e., closed, ventilated rooms, for customers to exclude from the general smoking ban; thus, smoking rooms may be created, while there is also lack of effective control measures for the smoking ban. As a result of legal possibility of creating separated smoking rooms, in 2015, there was a fourfold increase in the percentage of people who claimed that there was a room for smokers in their workplace. Finland is an example of a country where, similarly to Poland, it was possible to apply for a permission for smoking rooms; however, such solution was quickly recognized as insufficient protection against exposure to ETS, and regulations were introduced to strictly prohibit smoking in bars and restaurants [19,34,43]. In addition, research conducted in Finland, Australia, Canada, Germany, and Italy confirmed that only a total ban on smoking in hospitality venues protects against ETS exposure and leads to a decrease in the number of cigarettes smoked by employees [7,8,44,45,46,47,48].

The exposure to ETS of hospitality venues employees may be assessed with biomarker analysis (cotinine in saliva, urine, and plasma) or environmental monitoring (nicotine, PM2.5, and PM10), as well as an analysis of subjective feelings of employees (number of hours of ETS exposure) [49].

For the purposes of deepening the analysis, the authors of this study reviewed the literature and presented the analysis of publications on exposure biomarkers [49,50,51,52].

The biomarker for exposure to ETS used in all studies was cotinine, the main metabolite of nicotine. In the analysed studies, the reduction of the biomarker concentration in individuals exposed to ETS after the introduction of new legislative solutions compared to the concentration observed before the introduction amounted to 57–89% in establishments where the smoking ban was in force. For example, in studies conducted in Scotland, the concentration of cotinine in saliva before the introduction of new legislation was 2.9 ng/mL, while after the introduction, it decreased significantly to 0.4 ng/mL (by 89%, 95% CI: 85–92%). Fernandez et al. (2009) recorded a statistically significant decrease in the concentration of the exposure biomarker (from 1.6 to 0.5 ng/mL after the introduction of new legislative solutions; *p* < 0.01) (Table 7) only in hospitality venues where a total smoking ban was introduced [49,50,51,52,53,54,55,56,57].

## 5. Conclusions

1. Exposure to ETS among hospitality venues staff decreased in 2010–2015; however, it remained high.
  1.1. Despite the introduction of more restrictive regulations that prohibited smoking in hospitality venues (amendment to the Act on the Protection of Health against the Consequences of Use of Tobacco and Tobacco Products from 2010), more than half of nonsmoking employees were exposed to ETS in the workplace.
2. Regulations prohibiting smoking in hospitality venues were often violated by employees and customers.
  2.1. There is a need for effective control of the implementation and compliance with anti-tobacco laws in hospitality venues.
3. Only a total ban on smoking in all enclosed work spaces can serve as an appropriate protection of employees’ health (including employees of hospitality and commercial venues). Legislative solutions should be introduced to reduce exposure to ETS by placing a total ban on smoking.

## Figures and Tables

**Table 1:** The overview of the study group.

The Overview of the Study Group		2010		2015		Statistical Differences
		*N*	%	*N*	%	
Respondents’ age	20 years and less	234	17.2	136	10.9	*p* > 0.05
	21–30	867	63.7	838	67.2	
	31–40	178	13.1	175	14	
	41+	81	6.0	98	7.9	
Gender	F	885	65.1	810	65	*p* > 0.05
	M	475	34.9	437	35	
Education	Primary	17	1.2	15	1.2	*p* > 0.05
	Vocational	139	10.2	125	10	
	Secondary	810	59.6	733	58.8	
	Higher	394	29	374	30	
Marital status	Single	999	73.5	566	45.4	*p* > 0.001; chi^2^ = 272.655
	Married	334	24.6	498	39.9	
	Divorced	16	1.2	166	13.3	
	Widow (er)	11	0.8	17	1.4	

**Table 2:** Analysis of smoking by hospitality venues staff and their exposure to environmental tobacco smoke (ETS).

Analysis of Smoking and Exposure to ETS		2010		2015		Statistical Differences
		*N*	%	*N*	%	
Smoking among the staff	Every day	339	24.9	287	23	*p* < 0.01; chi^2^ = 14.472
	Sometimes	141	10.4	83	6.7	
	I do not smoke	880	64.7	877	70	
The number of cigarettes smoked per day by an employee	Up to 10	122	25.4	170	46	*p* < 0.01; chi^2^ = 212.883
	11–20	249	51.9	115	31	
	21–30	5	1	82	22.2	
	31+	1	0.2	3	0.8	
	I don’t know	103	21.5	–	–	
Workplace—type of room	Smoking room	423	31.1	59	4.7	*p* < 0.01; chi^2^ = 420.681
	Nonsmoking room	511	37.6	917	73.5	
	Both rooms	426	31.3	271	21.7	
Compliance with the smoking ban in nonsmoking rooms	The regulations are followed	1312	96.5	1194	88.5	*p* < 0.01; chi^2^ = 60.374
	The regulations are not followed	35	48	143	11.5	
Presence of a smoking room in the workplace	Yes	1222	89.9	698	56	*p* < 0.01; chi^2^ = 384.715
	No	138	10.1	549	44	
Staying in smoking rooms by nonsmoking employees	Every day	431	49	219	25	*p* < 0.001; chi^2^ = 120.907
	Once a week	8.5	75	90	10.3	
	Once a month	133	15.1	145	16.5	
	Never	241	27.4	423	48.2	

**Table 3:** Impact of selected factors which expose hospitality venues staff to ETS in the workplace in 2010—single-factor analysis.

Variable	Exposed *N* = 639		Not Exposed *N* = 241		One-Factor Logistic Regression 2010		
**Age**	***N***	**%**	***N***	**%**	**OR**	**95% CI**	***p***
20	174	89.2	21	10.8	7.87	3.48–17.82	***p*** **< 0.001**
21–25	257	76.3	80	23.7	3.05	1.48–6.30	***p*** **< 0.01**
26–30	128	59.8	86	40.2	1.41	0.68–2.94	*p* > 0.05
31–35	42	61.8	26	38.2	1.53	0.66–3.55	*p* > 0.05
36–40	18	66.7	9	33.3	1.90	0.66–5.44	*p* > 0.05
41+	20	51.3	19	48.7	1.00	Ref.	
**Gender**	***N***	**%**	***N***	**%**	**OR**	**95% CI**	***p***
Men	152	70.7	63	29.3	0.88	0.63–1.24	*p* > 0.05
Women	487	73.2	178	26.8	1.00	Ref.	
**Marital status**	***N***	**%**	***N***	**%**	**OR**	**95% CI**	***p***
Single	539	80.7	129	19.3	4.88	3.47–6.88	***p*** **< 0.001**
Married	89	46.1	104	53.9	1.00	Ref.	
Divorced	4	36.4	7	63.6	0.67	0.19–2.54	*p* > 0.05
Widow (er)	7	87.5	1	12.5	8.18	0.98–67.97	*p* > 0.05
**Education**	***N***	**%**	***N***	**%**	**OR**	**95% CI**	***p***
Primary and vocational	65	95.6	3	0.4	17.76	5.42–58.17	***p*** **< 0.001**
Secondary	441	77.4	129	22.6	2.80	2.03–3.86	***p*** **< 0.001**
Higher	133	55.0	109	45.0	1.00	Ref.	
**Position**	***N***	**%**	***N***	**%**	**OR**	**95% CI**	***p***
Owner/manager	2	0.2	123	98.0	1.00	Ref.	
Bartender/waiter	576	92.2	49	7.8	72.29	17.30–30.20	***p*** **< 0.001**
Cook	55	47.4	61	52.6	55.45	13.06–235.46	***p*** **< 0.001**
Other	6	42.9	8	57.1	46.13	7.97–266.85	***p*** **< 0.001**
**Is there a smoking room in the establishment**	***N***	**%**	***N***	**%**	**OR**	**95% CI**	***p***
Yes	591	73.6	212	26.4	1.68	1.03–2.74	***p*** **< 0.05**
No	48	62.3	29	37.7	1.00	Ref.	
**Is the anti-tobacco law followed**	***N***	**%**	***N***	**%**	**OR**	**95% CI**	***p***
Yes	609	71.8	239	28.2	1.00	Ref.	
No	30	93.8	2	6.2	5.89	1.39–24.88	***p*** **< 0.05**
**Room where the respondent works**	***N***	**%**	***N***	**%**	**OR**	**95% CI**	***p***
For smokers	210	87.5	30	12.5	6.80	4.38–10.54	***p*** **< 0.001**
For nonsmokers	173	50.7	168	49.3	1.00	Ref.	
Both rooms	256	85.6	43	14.4	5.78	3.92–8.52	***p*** **< 0.001**
**Is there a ban on smoking outside the room?**	***N***	**%**	***N***	**%**	**OR**	**95% CI**	***p***
Yes	375	72.5	142	27.5	1.00	Ref.	
No	264	72.7	99	27.3	1.01	0.73–1.39	*p* > 0.05
**Is the ban on smoking outside the smoking room observed?**	***N***	**%**	***N***	**%**	**OR**	**95% CI**	***p***
Yes	162	70.7	67	29.3	1.00	Ref.	
No	477	73.3	174	26.7	1.13	0.81–1.58	*p* > 0.05

**Table 4:** Impact of selected factors which expose hospitality venues staff to ETS in the workplace in 2015—single-factor analysis.

Variable	Exposed *N* = 454		Not Exposed *N* = 423		One-Factor Logistic Regression 2015		
**Age**	***N***	**%**	***N***	**%**	**OR**	**95% CI**	***p***
<20	72	52.6	65	47.4	1.10	0.55–2.25	*p* > 0.05
21–25	150	51.2	143	48.8	1.05	0.54–2.04	*p* > 0.05
26–30	154	52.0	142	48.0	1.08	0.56–2.11	*p* > 0.05
31–35	38	60.3	25	39.7	1.52	0.68–3.39	*p* > 0.05
36–40	19	42.3	27	58.7	0.70	0.30–1.66	*p* > 0.05
41+	21	50.0	21	50.0	1.00	Ref.	
**Gender**	***N***	**%**	***N***	**%**	**OR**	**95% CI**	***p***
Men	150	53.2	132	46.8	1.09	0.82–1.45	*p* > 0.05
Women	304	51.1	291	48.9	1.00	Ref.	
**Marital status**	***N***	**%**	***N***	**%**	**OR**	**95% CI**	***p***
Single	214	57.5	158	42.5	1.43	1.08–1.91	*p* < 0.05
Married	187	48.6	198	51.4	1.00	Ref.	
Divorced	48	44.9	59	55.1	0.86	0.56–1.33	*p* > 0.05
Widow (er)	5	38.5	8	61.5	0.66	0.21–2.06	*p* > 0.05
**Education**	***N***	**%**	***N***	**%**	**OR**	**95% CI**	***P***
Primary and vocational	50	58.1	36	41.9	0.90	0.54–1.51	*p* > 0.05
Secondary	260	47.0	293	53.0	0.58	0.43–0.79	*p* > 0.05
Higher	144	60.5	94	39.5	1.00	Ref.	
**Position**	***N***	**%**	***N***	**%**	**OR**	**95% CI**	***p***
Owner/manager	44	61.1	28	38.9	1.00	Ref.	
Bartender/waiter	343	50.5	336	49.5	1.54	0.94–2.53	*p* > 0.05
Cook	59	51.3	56	48.7	1.49	0.82–2.72	*p* > 0.05
Other	8	72.7	3	27.3	0.59	0.14–2.42	*p* > 0.05
**Is the anti-tobacco law followed**	***N***	**%**	***N***	**%**	**OR**	**95% CI**	***p***
Yes	389	48.5	413	51.5	1.00	Ref.	
No	65	86.7	10	13.3	6.90	3.49–13.63	***p*** **< 0.001**
**Is there a ban on smoking outside the room for customers**	***N***	**%**	***N***	**%**	**OR**	**95% CI**	***p***
Yes	358	46.2	417	53.8	1.00	Ref.	
No	96	94.1	6	5.9	18.64	8.06–43.08	***p*** **< 0.001**
**Is the ban on smoking outside the room for customers observed?**	***N***	**%**	***N***	**%**	**OR**	**95% CI**	***p***
Yes	355	46.7	405	53.3	1.00	Ref.	
No	99	84.6	18	15.4	6.27	3.72–10.58	***p*** **< 0.001**
**Type of establishment**	***N***	**%**	***N***	**%**	**OR**	**95% CI**	***p***
Restaurant/café	268	48.6	284	51.4	1.00	Ref.	
Bar	138	52.5	125	47.5	1.17	0.87–1.57	*p* > 0.05
Night club or music club	48	77.4	14	22.6	3.63	1.96–6.75	***p*** **< 0.001**
**Working hours**	***N***	**%**	***N***	**%**	**OR**	**95% CI**	***p***
Until 6:00 p.m.	279	52.0	258	48.0	1.00	Ref.	
After 6:00 p.m.	175	51.5	165	48.5	2.41	1.66–3.49	*p* < 0.001
**Is the respondent concerned about the effects of ETS on health**	***N***	**%**	***N***	**%**	**OR**	**95% CI**	***p***
Yes	395	54.3	333	45.7	1.00	Ref.	
No or not much	59	39.6	90	60.4	0.56	0.39–0.79	***p*** **< 0.01**
**Workplace smoking rules**	***N***	**%**	***N***	**%**	**OR**	**95% CI**	***p***
Smoking is allowed	46	83.6	9	16.4	9.68	4.54–20.65	***p*** **< 0.001**
Smoking is allowed in designated areas	112	64.7	61	35.3	3.48	2.33–5.19	***p*** **< 0.001**
Smoking is prohibited everywhere	94	34.6	178	65.4	1.00	Ref.	
Smoking is prohibited in rooms for customers	44	20.8	168	79.2	0.50	0.33–0.75	***p*** **< 0.001**
There is a separate smoking room and a nonsmoking room	158	95.8	7	4.2	42.74	19.24–94.97	***p*** **< 0.001**
**Is there a designated smoking room in the establishment**	***N***	**%**	***N***	**%**	**OR**	**95% CI**	***p***
Yes	310	67.1	152	32.9	3.84	2.90–5.08	***p*** **< 0.001**
No	144	34.7	271	65.3	1.00	Ref.	
**Is there a policy for employees**	***N***	**%**	***N***	**%**	**OR**	**95% CI**	***p***
Yes	312	48.7	329	51.3	1.00	Ref.	
No	31	75.6	10	24.4	3.27	1.57–6.79	***p*** **< 0.01**
I don’t know	111	56.9	84	43.1	1.39	1.01–1.93	***p*** **< 0.05**
**Was the policy communicated to employees**	***N***	**%**	***N***	**%**	**OR**	**95% CI**	***p***
Yes	225	44.3	283	55.7	1.00	Ref.	
No/I don’t know	229	62.1	140	37.9	2.06	1.56–2.71	***p*** **< 0.001**
**Is there a policy for customers**	***N***	**%**	***N***	**%**	**OR**	**95% CI**	***p***
Yes	345	47.9	376	52.1	1.00	Ref.	
No	34	82.9	7	17.1	5.29	2.31–12.11	***p*** **< 0.001**
I don’t know	75	65.2	40	34.8	2.04	1.35–3.08	***p*** **< 0.001**
**No smoking signs in the establishment**	***N***	**%**	***N***	**%**	**OR**	**95% CI**	***p***
Yes	410	50.4	403	49.6	1.00	Ref.	
No	13	68.4	6	31.6	2.13	0.80–5.67	*p* > 0.05
I don’t know	31	68.9	14	31.1	2.18	1.14–4.16	***p*** **< 0.05**
**Exposure to ETS causes heart disease in nonsmokers**	***N***	**%**	***N***	**%**	**OR**	**95% CI**	***p***
I don’t agree	63	77.8	18	22.2	3.63	2.11–6.24	***p*** **< 0.001**
I agree	391	49.1	405	50.9	1.00	Ref.	
**Exposure to ETS causes cancer in nonsmokers**	***N***	**%**	***N***	**%**	**OR**	**95% CI**	***p***
I don’t agree	81	77.9	23	22.1	3.78	2.33–6.13	***p*** **< 0.001**
I agree	373	48.3	400	51.7	1.00	Ref.	

**Table 5:** Impact of selected factors which expose hospitality venues staff to ETS in the workplace in 2010—multifactor analysis.

Variable	Exposed *N* = 639		Not Exposed *N* = 241		Multifactor Logistic Regression 2010		
**Age**	***N***	**%**	***N***	**%**	**OR**	**95% CI**	***p***
<20	174	89.2	21	10.8	1.08	0.43–2.76	*p* > 0.05
21–25	257	76.3	80	23.7	0.76	0.19–1.03	*p* > 0.05
26–30	128	59.8	86	40.2	0.43	0.19–0.99	0.05
31–35	42	61.8	26	38.2	1.01	0.39–2.59	*p* > 0.05
36–40	18	66.7	9	33.3	1.28	0.38–4.29	*p* > 0.05
41+	20	51.3	19	48.7	1.00	Ref.	
**Marital status**	***N***	**%**	***N***	**%**	**OR**	**95% CI**	***p***
Single	539	80.7	129	19.3	3.86	2.47–6.03	***p*** **< 0.001**
Married	89	46.1	104	53.9	1.00	Ref.	
Divorced	4	36.4	7	63.6	0.03	0.01–0.20	*p* > 0.05
Widow(er)	7	87.5	1	12.5	8.47	0.93–77.59	*p* > 0.05
**Education**	***N***	**%**	***N***	**%**	**OR**	**95% CI**	***p***
Primary + vocational + secondary	506	79.3	132	20.7	4.78	3.10–7.37	***p*** **< 0.001**
Higher	133	55.0	109	45.0	1.00	Ref.	
**Is there a smoking room in the establishment**	***N***	**%**	***N***	**%**	**OR**	**95% CI**	***p***
Yes	591	73.6	212	26.4	0.96	0.51–1.81	*p* > 0.05
No	48	62.3	29	37.7	1.00	Ref.	
**Is the anti-tobacco law followed**	***N***	**%**	***N***	**%**	**OR**	**95% CI**	***p***
Yes	609	71.8	239	28.2	1.00	Ref.	
No	30	93.8	2	6.2	24.73	3.54–172.88	***p*** **< 0.001**
**Room where the respondent works**	***N***	**%**	***N***	**%**	**OR**	**95% CI**	***p***
For smokers	210	87.5	30	12.5	11.10	6.50–18.94	***p*** **< 0.001**
For nonsmokers	173	50.7	168	49.3	1.00	Ref.	
Both rooms	256	85.6	43	14.4	7.05	4.33–11.48	***p*** **< 0.001**

**Table 6:** Impact of selected factors which expose hospitality venues staff to ETS in the workplace in 2015—multifactor analysis.

Variable	Exposed *N* = 454		Not Exposed *N* = 423		Multifactor Logistic Regression 2015		
**Age**	***N***	**%**	***N***	**%**	**OR**	**95% CI**	***p***
<20	72	52.6	65	47.4	1.01	0.31–3.25	*p* > 0.05
21–25	150	51.2	143	48.8	1.27	0.44–3.69	*p* > 0.05
26–30	154	52.0	142	48.0	1.67	0.58–4.80	*p* > 0.05
31–35	38	60.3	25	39.7	2.63	0.80–8.71	*p* > 0.05
36–40	19	42.3	27	58.7	2.81	0.81–9.75	*p* > 0.05
41+	21	50.0	21	50.0	1.00	Ref.	
**Marital status**	***N***	**%**	***N***	**%**	**OR**	**95% CI**	***p***
Single	214	57.5	158	42.5	1.41	0.91–2.18	*p* > 0.05
Married	187	48.6	198	51.4	1.00	Ref.	
Divorced	48	44.9	59	55.1	1.20	0.66–2.19	*p* > 0.05
Widow(er)	5	38.5	8	61.5	1.14	0.27–4.81	*p* > 0.05
**Is the anti-tobacco law followed**	***N***	**%**	***N***	**%**	**OR**	**95% CI**	***p***
Yes	389	48.5	413	51.5	1.00	Ref.	
No	65	86.7	10	13.3	0.50	0.13–1.83	*p* > 0.05
**Is there a ban on smoking outside the restaurant room**	***N***	**%**	***N***	**%**	**OR**	**95% CI**	***p***
Yes	358	46.2	417	53.8	1.00	Ref.	
No	96	94.1	6	5.9	3.53	1.55–8.04	***p*** **< 0.01**
**Is the ban on smoking outside the room observed?**	***N***	**%**	***N***	**%**	**OR**	**95% CI**	***p***
Yes	355	46.7	405	53.3	1.00	Ref.	
No	99	84.6	18	15.4	6.63	2.05–21.54	***p*** **< 0.01**
**Working hours**	***N***	**%**	***N***	**%**	**OR**	**95% CI**	***p***
Until 6:00 p.m.	279	52.0	258	48.0	1.00	Ref.	
After 6:00 p.m.	175	51.5	165	48.5	0.62	0.34–1.15	*p* > 0.05
**Is the respondent concerned about the effects of ETS on health**	***N***	**%**	***N***	**%**	**OR**	**95% CI**	***p***
Yes	395	54.3	333	45.7	1.00	Ref.	
No or not much	59	39.6	90	60.4	0.30	0.16–0.56	***p*** **< 0.001**
**Workplace smoking rules**	***N***	**%**	***N***	**%**	**OR**	**95% CI**	***p***
Smoking is allowed	46	83.6	9	16.4	0.81	0.20–3.36	*p* > 0.05
Smoking is allowed in designated areas	112	64.7	61	35.3	1.87	0.97–3.63	*p* > 0.05
Smoking is prohibited everywhere	94	34.6	178	65.4	1.00	Ref.	
Smoking is prohibited in rooms for customers	44	20.8	168	79.2	0.58	0.34–0.99	***p*** **< 0.05**
There is a separate smoking room and a nonsmoking room	158	95.8	7	4.2	10.88	3.94–30.03	***p*** **< 0.001**
**Is there a designated smoking room in the establishment**	***N***	**%**	***N***	**%**	**OR**	**95% CI**	***p***
Yes	310	67.1	152	32.9	1.38	0.79–2.41	*p* > 0.05
No	144	34.7	271	65.3	1.00	Ref.	
**Is there a policy for employees**	***N***	**%**	***N***	**%**	**OR**	**95% CI**	***p***
Yes	312	48.7	329	51.3	1.00	Ref.	
No/I don’t know	142	60.2	94	39.8	5.11	1.99–13.15	***p*** **< 0.001**
**Was the policy communicated to employees**	***N***	**%**	***N***	**%**	**OR**	**95% CI**	***p***
Yes	225	44.3	283	55.7	1.00	Ref.	
No/I don’t know	229	62.1	140	37.9	1.96	1.25–3.06	***p*** **< 0.01**
**Is there a policy for customers**	***N***	**%**	***N***	**%**	**OR**	**95% CI**	***p***
Yes	345	47.9	376	52.1	1.00	Ref.	
No	109	69.9	47	30.1	1.45	0.49–4.25	*p* > 0.05
**No smoking signs in the establishment**	***N***	**%**	***N***	**%**	**OR**	**95% CI**	***p***
Yes	410	50.4	403	49.6	1.00	Ref.	
No	13	68.4	6	31.6	1.21	0.24–6.02	*p* > 0.05
I don’t know	31	68.9	14	31.1	1.84	0.60–5.64	*p* > 0.05
**Exposure to ETS causes heart disease in nonsmokers**	***N***	**%**	***N***	**%**	**OR**	**95% CI**	***p***
I don’t agree	63	77.8	18	22.2	1.72	0.72–4.08	*p* > 0.05
I agree	391	49.1	405	50.9	1.00	Ref.	
**Exposure to ETS causes cancer in nonsmokers**	***N***	**%**	***N***	**%**	**OR**	**95% CI**	***p***
I don’t agree	81	77.9	23	22.1	7.95	3.64–17.34	***p*** **< 0.001**
I agree	373	48.3	400	51.7	1.00	Ref.	

**Table 7:** Changes in the level of ETS exposure after the implementation of legislative smoking bans—analysis based on biomarkers of exposure.

No.	Country	Characteristic			Reduction	References
1.	Ireland	Legislation (date of implementation and type of restrictions)		III 2004 ban of smoking in indoor workplaces	80% (*p* < 0.001)	Allwright, 2005 [49], Mulcahy, 2005 [50]
		Populations		111 bar staff		
		Biomarker of exposure		Cotinine level in saliva Md (IQR)		
		Biomarker level	Before smoking ban	29.0 nmol/L (18.2–43.2 nmol/L)		
			After smoking Ban	5.1 nmol/L (2.8–13.1 nmol/L)		
2.	Norway	Legislation (date of implementation and type of restrictions)		Ban on smoking in indoor workplaces	*p* < 0.001	Ellingsen, 2006 [51]
		Populations		25 employees in bars and restaurants		
		Biomarker of exposure		Cotinine level in urine GM (95% CI)		
		Biomarker level	Before smoking ban	Evening urine collection: 9.5 μg/g (6.5–13.7 μg/g) creatinine/Morning urine collection: 15.3 μg/g (10.3–22.7 μg/g) creatinine		
			After smoking Ban	Evening urine collection: 1.4 μg/g (0.8–2.5 μg/g) creatinine/Morning urine collection: 1.6 μg/g (0.9–3.0 μg/g) creatinine	*p* < 0.001	
3.	Italy	Legislation (date of implementation and type of restrictions)		I 2005 ban on smoking in indoor workplaces	*p* < 0.0001	Valente, 2007 [52]
		Populations		37 hospitality workers		
		Biomarker of exposure		Cotinine level in urine GM (95% CI)		
		Biomarker level	Before smoking ban	15.4 ng/mL (913–18.3 ng/mL)		
			After smoking ban	2.6 ng/mL (1.4–4.9 ng/mL)		
4.	Sweden	Legislation (date of implementation and type of restrictions)		VI 2005 ban on smoking in indoor workplaces	bd.	Larsson, 2008 [53]
		Populations		43 hospitality workers		
		Biomarker of exposure		Cotinine level in urine/percentage of people with cotinine level below the limit of detection		
		Biomarker level	Before smoking ban	37%		
			After smoking ban	67%		
5.	Scotland	Legislation (date of implementation and type of restrictions)		III 2006 ban on smoking in indoor workplaces	89% (85–92%)	Semple, 2007 [54], Menzies, 2006 [55]
		Populations		126 hospitality workers		
		Biomarker of exposure		Cotinine level in saliva GM (GSD)		
		Biomarker level	Before smoking ban	2.9 ng/mL (2.3 ng/mL)		
			After smoking ban	0.4 ng/mL (3.7 ng/mL)		
6.	England	Legislation (date of implementation and type of restrictions)		Ban on smoking in indoor workplaces	75% (*p* < 0.001)	Gotz, 2008 [56]
		Populations		75 workers		
		Biomarker of exposure		Cotinine level in saliva M (GM; SD)		
		Biomarker level	Before smoking ban	3.4 ng/mL (2.4 ng/mL; 2.5 ng/mL)		
			After smoking ban	0.8 ng/mL (0.4 ng/mL; 3.2 ng/mL)		

GSD—geometric standard deviation; Md—mediana; SD—standard deviation; IQR—interquartile range; bd.—no data. Źródło: Polańska, K.; Hanke, W.; Konieczko, K. Hospitality workers’s exposure to ETS before and after implementation of smoking ban in public places: a review of epidemiological studies. Medycyna Pracy 2011, 62, 211–224 [57].
